# Endovascular embolization of pancreatic arteriovenous malformation associated with acute pancreatitis

**DOI:** 10.1093/jscr/rjaf775

**Published:** 2025-09-29

**Authors:** Jidong Yang, Qingliang Zhu, Hailong Zhang

**Affiliations:** Vascular Interventional Department, JingMen People's Hospital, JingMen, China; Department of Gastroenterology, The Affiliated Hospital of Southwest Medical University, Luzhou, China; Department of Gastroenterology, The Affiliated Hospital of Southwest Medical University, Luzhou, China

**Keywords:** pancreatic arteriovenous malformation, acute pancreatitis, transarterial embolization

## Abstract

Acute pancreatitis caused by pancreatic arteriovenous malformation is a rare entity that has no established guideline for treatment. We present a 45-year-old man with upper abdominal pain diagnosed as acute pancreatitis resulting from pancreatic arteriovenous malformation. Transarterial embolization was successful in treating pancreatic arteriovenous malformation, the clinical outcome of the patient was good with no recurrent acute pancreatitis or abdominal pain for 32 months since the embolization.

## Introduction

Pancreatic arteriovenous malformation (PAVM) is a rare vascular anomaly which can be asymptomatic or causes portal hypertension, gastrointestinal bleeding, abdominal pain, and acute pancreatitis (AP), while AP is less common. Because only few cases are reported to date, there are no universally accepted guidelines for the management of PAVM with AP. Surgery is the most reported treatment and considered to be the ultimate treatment, endovascular treatment also has been reported to be an alternative management in selective patients, but the cases are very few. We report a case of patient who received endovascular embolization for control of PAVM with AP and a literature review is also presented. The Institutional Review Board waived informed consent for this retrospective study.

## Case report

**Figure 1:**
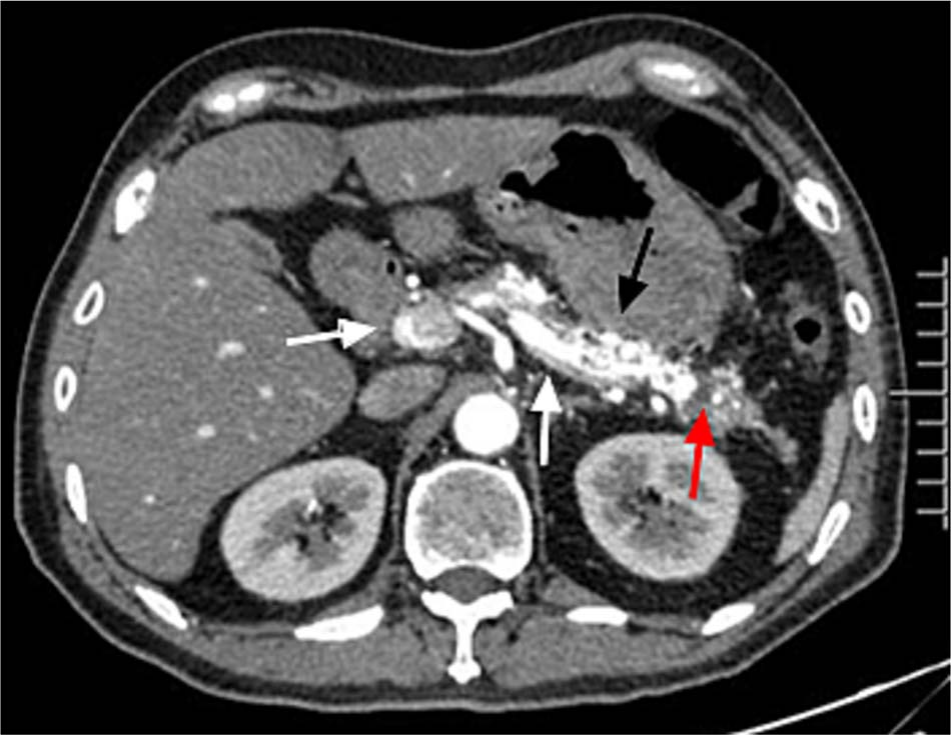
Enhanced CT showed tortuous dilated arteries in the pancreas body and tail (black arrow) with a small pseudocyst (red arrow) and early opacification of the splenic vein and portal vein (white arrows).

A 45-year-old man with upper abdominal pain for 3 days was referred to our unit. His past history was unremarkable. Upon physical examination, He had abdominal tenderness. Routine blood test showed elevated amylase and lipase (395.8 and 536.7 U/l). Enhanced computed tomography (CT) demonstrated tortuous dilated arteries in the pancreas body and tail with a small pseudocyst, and early opacification of the portal vein, but no signs of portal hypertension (Fig. 1). Endoscopy revealed no varices and ulcers. Based on these findings, a diagnosis of PVAM with AP was made.

**Figure 2:**
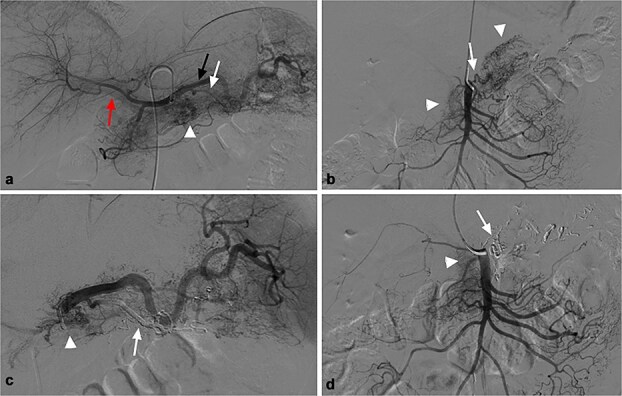
(a) Celiac angiography showed PVAM (white arrowhead) with numerous fine feeders mainly originating from splenic artery (black arrow) and great pancreatic artery (white arrow), as well as early portal vein opacification (red arrow). (b) Superior mesenteric angiography shows other fine feeders mainly originating from one branch of superior mesenteric (white arrow) artery with PVAM (white arrowhead). (c) Post embolization angiogram of splenic artery shows occlusion of great pancreatic artery (white arrow), and minimal residual staining of the pancreas (white arrowhead). (d) Post embolization angiogram of the supplying branch shows occlusion of the concerned artery (white arrow) and minimal residual staining of the pancreas (white arrowhead).

**Figure 3:**
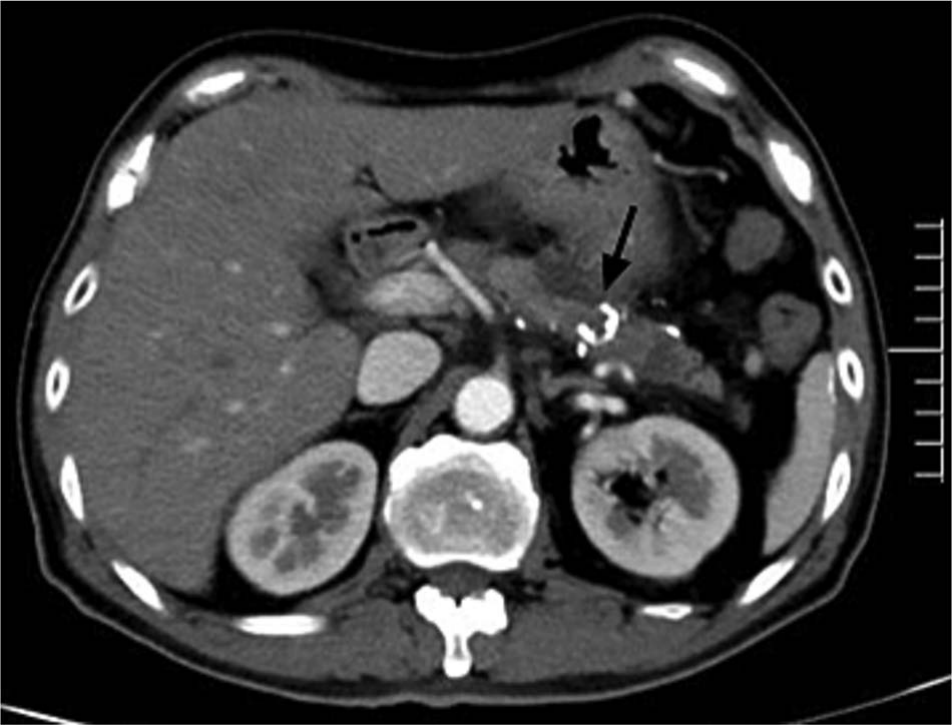
Postembolic enhanced CT after 7 months showed disappearance of PAVM with high-density spots (NBCA) in the pancreas (black arrow).

For further diagnosis and treatment, angiography and embolization under local anesthesia were agreed on. Celiac and superior mesenteric angiography through the right femoral approach confirmed PVAM which mainly originated from great pancreatic artery (Fig. 2a) and superior mesenteric artery (Fig. 2b). Selective embolization of great pancreatic artery was performed using a mixture of *n*-butyl-2-cyanoacrylate (NBCA) (Histoacryl; Compont, Beijing, China) and iodized oil (Poppy Ethidium, Hengrui, Jiangsu, China) combined in a 1:3 ratio. After the embolization, repeat angiography showed significant regression of the PVAM (Fig. 2c). Because selectively catheterizing the major feeding branch of superior mesenteric artery failed through the right femoral approach, embolization using the same mixture was done through the right radial approach. After the embolization, only minimal residual staining of the pancreas can be seen (Fig. 2d). The clinical outcome was good, the patient suffered mild abdominal pain for 2 days and no severe embolization-related complications, and was discharged 4 days after the procedure. Follow-up CT scan after 8 months demonstrated progressive regression of the PAVM (Fig. 3). At the time of writing this manuscript, the patient had completed 32 months of follow-up and had no recurrent AP or abdominal pain.

## Discussion

AP is an uncommon complication of PVAM, the prevalence is not clear because most of cases were reported sporadically. We searched PubMed from January 1980 to December 2022 using the keywords “pancreatic arteriovenous malformation” and “pancreatitis,” 18 cases about AP caused by PVAM are reported in English literature, while only four of them are treated by endovascular treatment (Table 1) [1–4].

**Table 1:** Summary of reported cases of PAVM associated with AP treated by endovascular embolization

Author/reference, year	Age/sex	Site	Image	Embolic materials	Prognosis
Yoon [1], 2020	43/M	Pancreatic body and tail	CT/MRI	Microcoils, gelatin sponges, histoacryl glue	Good
Rajesh [2], 2016	46/M	Pancreatic head and body	CT	Histoacryl glue	Good
Frenk [3], 2016	54/M	Pancreatic head and body	CT	gelatin sponges, ethanol	Good
Cassinotto [4], 2015	56/M	Pancreatic head	CT	Onyx	Good

The mechanisms of AP caused by PVAM may include pancreatic ductal compression caused by PAVM, pooling of blood in the pancreatic duct, or ischemia as a result of vascular steal [5]. Because the symptoms are not specific, PVAM is usually suspected by imaging. As demonstrated by Table 1, enhanced CT and MRI play an important role in detecting PVAM and AP. The characteristic features of P-AVM on CT include conglomeration of strong nodular stains in the pancreas and early contrast filling of the portal vein during the arterial phase. Angiography is the golden criteria when suspecting PVAM. The angiographic findings of PAVM are characterized by tortuous dilated arteries in the pancreas and early opacification of portal vein.

Although surgery is a definitive treatment for PAVM, but we should consider the possibility of pancreatic fistula, bleeding, diabetes, and so on caused by pancreatectomy or contraindications to surgery [6, 7]. Endovascular treatment has also been reported to be used in the management of PAVM with portal hypertension or gastrointestinal bleeding, but it has a high failure rate [8, 9]. The reason may be that there are more feeders and arterial blood filling into portal system compared to patients with pancreatitis, it’s usually impossible to embolize all feeders, and residual feeders or secondary collateral pathways still can cause recurrence of portal hypertension, while residual fine feeders may gradually decrease in patients with AP [1, 2, 10], as in our case.

Preembolic imaging and angiography are integral to choose appropriate embolic materials and avoid ectopic embolization. Different embolic materials for AVM embolization such as coils, gelatin sponge, poly-vinyl-alcohol particles, NBCA, and ethylene vinyl alcohol copolymer (Onyx) have been reported [3, 10], but the most suitable embolic materials have not been established due to the lack of cases. Permanent embolic materials are recommended by many to be used to prevent recurrence of AVM, and liquid embolic materials are undoubtedly more effective because they allow more efficient filling of the PAVM and obliteration of the vascular bed [4]. The most used liquid embolic materials are NBCA and Onyx, but which one is safer and more effective remains controversial. In our case, NBCA was successfully applied with obliteration of the vascular bed in the pancreas body and tail, and with no reported complication related to embolization.

## Conclusion

In our case, endovascular embolization of PAVM associated with AP proved to be safe and effective, this technique may be an alternative option for selective patients.
